# Analysis of ART effects and drug resistance in adult HIV/AIDS patients in Meigu County, Liangshan Prefecture, China

**DOI:** 10.1186/s12879-024-09048-y

**Published:** 2024-02-01

**Authors:** Li Yuan, Kaiyou Chen, Yuanfang Cai, Zhonghui Zhou, Ju Yang, Wuti Jiqu, Qirong Zhu, Hong Zhang, Shaowei Niu, Hui Sun

**Affiliations:** 1 Affiliated Hospital of North Sichuan Medical College, Nanchong, Sichuan China; 2 Meigu County People’s Hospital, Meigu, Liangshan Prefecture, Sichuan China

**Keywords:** AIDS, Antiretroviral therapy, Drug resistance

## Abstract

**Background objective:**

This study aimed to understand the basic situation of adults with human immunodeficiency virus (HIV) receiving antiretroviral therapy (ART) in Meigu County, Liangshan Yi Autonomous Prefecture. The information of patients who had been on ART for more than 6 months, the effect of ART, the possible reasons for ART failure, knowledge of drug resistance among patients with ART failure and the possible reasons for the emergence of drug resistance were analyzed.

**Methods:**

A total of 2753 people living with HIV (PLWH) were collected for HIV-1 RNA virus nucleic acid testing. Plasma specimens with HIV-1 RNA ≥ 1000 copies/mL were sent to the laboratory for nucleic acid extraction, PCR, electrophoresis and sequencing, and the sequencing results were submitted to the HIV drug resistance database of Stanford University for subtyping to determine the drug resistance mutation sites and drug sensitivity levels.

**Results:**

A total of 2753 patients were enrolled in this study. Antiviral therapy failed in 288 patients and was successfully amplified in 245, of which 111 had resistance genes. The resistance rate to failure of viral suppression was 45.3% (111/245). The highest rates of resistance to NNRTIs were found for efavirenz (EFV) and nevirapine (NVP) (42.9%), and the highest rates of resistance to NRTIs were found for 3TC and emtricitabine (FTC) (15.9%). The most common NNRTI resistance mutation site was K103N (20.8%), followed by V179D (9.4%) and V106M (7.8%); the most common NRTI resistance mutation site was M184V/I/MV (14.3%), followed by K65R (6.9%); three PI-associated resistance mutation sites were identified. The subtype of the resistant strain was CRF07-BC in almost all patients (98.9%).

**Conclusions:**

Compared with the previous low ART efficacy in the county, this study showed that the overall virological failure (VF) resistance rate in the county is still low, dominated by resistance to EFV, NVP, 3TC, FTC, and didanosine (DDI). Due to economic constraints, the core regimen is still 3TC + TDF, but before initiating ART, testing for HIV-1 subtypes and resistance should be conducted to avoid resistance that can lead to VF, especially for patients with high risk factors for resistance as shown by epidemiologic investigations.

## Background

Acquired immunodeficiency syndrome (AIDS) is a chronic infectious disease caused by human immunodeficiency virus (HIV). At present, antiretroviral therapy (ART) in China has made great progress, but in some remote mountainous areas, free drugs provided by the state are still used as the main medications, and with the prolongation of medication use, these drugs have an increased risk of drug resistance, which reduces the therapeutic effect and makes it difficult to achieve the goal of 95% HIV/AIDS control. Therefore, this study focused on Meigu County in Liangshan Prefecture, Sichuan Province, China, which is dominated by the Yi ethnic group, is a high-incidence area of the AIDS epidemic, and has a low economic development level. This study analyzed HIV-1 RNA, HIV-1 RNA resistance genes, and resistance-related risk factors in patients aged 18 years and older who had been on regular ART for more than 6 months, with the aim of providing a reference for the future development of ART regimens in this area.

## Methods

### Study participants

Medical staff called all candidate participants in the district using ART to introduce them to this study and to confirm their eligibility for participation. All participants signed an informed consent form prior to enrollment. Finally, a total of 2753 people living with HIV (PLWH) were enrolled in this study. A total of 2753 plasma samples were collected from HIV/AIDS patients aged 18 years and older in the district who had received ART for ≥6 months from January 1, 2010, to January 1, 2021, and the plasma was derived from specimens tested annually at regular intervals.

### Source

Data were obtained from the Chinese Center for Disease Control and Prevention Integrated AIDS Prevention and Control Information System, including patient name, sex, age, education level, marital status, family type, HIV transmission route, first CD4^+^ counts, treatment duration, and treatment regimen.

### Collection, preservation and transportation of plasma

Two milliliters of peripheral venous blood from the subject was drawn using a sterile syringe and injected into a sterile collection tube containing ethylenediaminetetraacetic acid (EDTA), and the patient’s plasma was separated by centrifugation at 1600 rpm for 5–20 minutes. Fresh plasma was drawn and injected into another sterile centrifuge tube for backup, and the sample could be used immediately for testing. The samples could be stored at − 70 °C for a long time for backup, avoiding repeated freezing and thawing, and were transported in a jug with ice or a foam box with ice after sealing.

### Experimental steps

#### Viral load detection

Viral load (VL) was detected in the plasma of the study subjects using a fully automated nucleic acid extractor AU1001–96 (Wuxi Biotec Biotechnology Co., Ltd.), and the performance of the assay was strictly in accordance with the instruction manual of the assay system. Virological failure (VF) was defined as a viral load ≥1000 copies/ml in an individual on ART for more than 6 months [1]. Plasma samples from VF patients were selected for the next step of genotypic drug resistance monitoring.

#### Genetic resistance testing

HIV genotypic resistance testing was performed on plasma with a viral load ≥1000 cp/ml using a laboratory self-constructed assay (in-house).

- ① Nucleic acid extraction: RNA was extracted using a MagNA Pure LC 2.0 fully automated nucleic acid extractor and MagNA Pure LC Total Nucleic Acid Isolation Kit, which is a high-performance reagent kit [Roche (Switzerland)].
- ② PCR: Using the extracted RNA as a template, the HIV-1 pol gene region was amplified by nested PCR with a length of 1200 bp, including the protease and reverse transcriptase regions, and the first round of PCR was performed using the AccessQuick RT–PCR System (A1702) kit (ProMag (Beijing) Biotechnology Co. Ltd.]); the above two PCR products were amplified using an Eppendorf 5333–47,507 PCR instrument (Eppendorf (Germany)).
- ③ Electrophoresis and sequencing: The PCR products were amplified by 1% agarose gel electrophoresis using a capillary electrophoresis instrument (QIAxcel Advanced, Germany) to confirm the correct molecular weight of the amplified fragments, and then the amplified products were sent to Beijing Noce Genome Research Center Co. The amplification and sequencing primers are shown in Table 1.

**Table 1:** Amplification and sequencing primers

Name	Sequence (5′-3′)	HXB2 position	Amplified fragment length (bp)
Round 1 PCR			
Upstream primer MAW	TTGGAAATGTGGAAAGGAAGGAC	2028 ~ 2050	151,226
Downstream primer RT21	CTGTATTTCTGCTATTAAGTCTTTTGATGGG	3509 ~ 3539	
Round 2 PCR			
Upstream primer PRO-	CAGAGCCAACAGCCCCACCA	2147 ~ 2166	11,811
Downstream primer RT4R	CTTCTGTATATCATTGACAGTCCAGCT	3299 ~ 3327	
Forward sequencing primers			
PROS3	GCCAACAGCCCCACCA	2151 ~ 2166	1176
RTAS	CTCAGATTGGTTGCAC	2524 ~ 2539	803
RT-B	CCTAGTATAAACAATGAGACAC	2946 ~ 2967	381
Reverse sequencing primers			
PROC1S	GCTGGGTGTGGTATTCC	2826 ~ 2842	695
RT4R	CTTCTGTATATCATTGACAGTCCAGCT	3299 ~ 3327	1180

### Definition of indicators

Referring to the 2013 WHO “Comprehensive guidelines for the use of antiretroviral drugs in the treatment and prevention of HIV infection”, the drug resistance rate = the number of resistant drugs/(number of investigations - number of amplification negatives)*100%, and the rate of resistance to viral suppression failure = the number of resistant drugs/(number of viral suppression failures - number of amplification negatives)*100%.

### Sequence analysis

The measured sequences were edited and spliced by ChromasPro 1.33 software and corrected by BioEdit software(Ibis Biosciences, Carlsbad, CA, USA), and the spliced sequences were submitted to the Stanford HIV drug resistance database (http://hivdb.stanford.edu) for subtyping to determine the drug resistance mutation sites and drug sensitivity levels. The Stanford University (HIVDB) system is categorized into five degrees of drug resistance according to the degree of drug resistance: sensitive (S), potentially resistant (P), low resistant (L), moderately resistant (I), and highly resistant (H) (https://hivdb.stanford.edu/).

### Statistical processing

The sociodemographic information of all study subjects was summarized, organized and analyzed using an Excel sheet, and SPSS 25.0 was used for descriptive and statistical analyses. Indicators such as the rate, composition ratio, mean and standard deviation were used to describe the basic information of AIDS patients. The variables were first subjected to a one-way chi-squared (χ^2^) test, and the group of variables with *P* < 0.05 was included in the multivariate logistic regression. The samples with virological failure and successful amplification were divided into drug-resistant and nondrug-resistant groups, and the variables were first compared between the two groups by the one-way chi-squared (χ^2^) test or Fisher’s exact test for statistical significance. The variables with *P* < 0.05 were again analyzed by multivariate logistic regression for the influence of the occurrence of drug resistance in patients, with a difference with *P* < 0.05 considered statistically significant.

## Results

### Sociodemographic information

As of January 1, 2021, there were 2753 HIV/AIDS patients aged 18 years and older who had been on ART for more than 6 months in Meigu County. Among them, 1697 (61.6%) were male; 2609 (94.8%) were aged 18–49 years; 2064 patients had not attended school (75.0%); 1834 had a spouse (66.6%); sexual transmission and intravenous drug transmission accounted for half each; first CD4^+^ counts of under 200 counts/μl occurred in 381 cases (13.8%), 200–349 counts/μl in 1787 cases (28.6%), 350–499 counts/μl in 1721 cases (26.2%), and 500 counts/μl and above in 864 cases (31.4%); an initial ART regimen with tenofovir disoproxil fumarate+lamivudine (TDF + 3TC) as the core drug was used in 2541 patients (93.3%) and non-TDF + 3TC as the core drug in 212 patients (7.7%); and the time on ART was 6 months-2 years for 1720 patients (62.5%), and the remaining patients were treated for more than 2 years (37.5%, 1033/2753). Specific information is shown in Table 2.

**Table 2:** Sociodemographic information of patients with a confirmed AIDS diagnosis in Meigu County

	Characteristic	Frequency (cases)	Composition ratio(%)
Sex	Male	1697	61.6
	Female	1056	38.4
Age group	18–49 years old	2609	94.8
	50–64 years old	136	4.9
	65 years old and above	8	0.3
Education level	Illiterate	2064	75.0
	Elementary school	498	18.1
	Junior high school	146	5.3
	High school or junior college	32	1.2
	College and above	13	0.5
Marital status	Unmarried	587	21.3
	Married	1834	66.6
	Divorced or widowed	332	12.1
Mode of transmission	Sexual transmission	1394	50.6
	Injection drugs	1359	49.4
Family type	Single family	867	31.5
	Extended family	857	31.1
	Other	1029	37.4
the first CD4+ counts counts/μl	below 200	381	13.8
	200–349	787	28.6
	350–499	721	26.2
	500 and above	864	31.4
initial ART regimen			
TDF + 3TC regimen		2541	93.3
	TDF + 3TC + EFV	2499	90.8
	TDF + 3TC + NVP	16	0.6
	TDF + 3TC + LPV/r	26	0.9
Non-TDF + 3TC regimen		212	7.7
	3TC + AZT + EFV	79	2.9
	3TC + AZT + NVP	43	1.6
	3TC + AZT + LPV/r	53	1.9
	3TC + D4T + NVP	34	1.2
	DDI + 3TC + EFV	3	0.1
time on ART (years)	6 months-2 years	1720	62.5
	3–5 years	602	21.9
	6–8 years	258	9.4
	≥9 years	173	6.3

### Initial ART regimen

The primary ART regimen in the district is the standard first-line regimen of the National Antiviral Program (TDF or AZT + 3TC + EFV/NVP), with rare use of TDF or AZT + 3TC + LPV/r regimens or other antiviral drugs.

### Analysis of the causes of virological failure

One-way chi-square (χ^2^) tests or Fisher’s exact tests were used to analyze the effect of patient sex, mode of transmission, first CD4^+^ counts, initial ART regimen, and time on ART, which were significantly associated with the risk of VF, on ART (*P* < 0.05), as shown in Table 3.

**Table 3:** One-way chi-square test of the factors influencing the failure of antiviral treatment

	Variable	Failure(%)	Success(%)	*χ*^2^	*P-value*
Sex	Female	78(7.4)	978(92.6)	17.29	<0.01
	Male	210(12.4)	1487(87.6)		
Age	18–49 years	276(10.6)	2333(89.4)	2.93	0.22
	50–64 years	12(8.8)	124(91.2)		
	65 years and older	0	8(100)		
Education level	Illiterate	217(10.5)	1847(89.5)	3.06	0.55
	Elementary school	50(10.0)	448(90.0)		
	Junior high school	19(13.0)	127(87.0)		
	High school or secondary school	1(3.1)	31(96.9)		
	College and above	1(7.7)	12(92.3)		
Marital status	Unmarried	68(11.6)	519(88.4)	1.07	0.59
	Married	185(10.1)	1649(89.9)		
	Divorced or widowed	35(10.5)	297(89.5)		
Mode of transmission	Sexual transmission	124(8.9)	1270(91.1)	7.39	<0.01
	Injection drugs	164(12.1)	1195(87.9)		
Family type	Single family	96(11.1)	771(88.9)	0.51	0.78
	Extended family	87(10.2)	770(89.8)		
	Other	105(10.2)	924(89.8)		
the first CD4+ counts counts/μl	Below 200	51(13.4)	330(86.6)	14.25	<0.01
	200–349	98(12.5)	689(87.5)		
	350–499	73(10.1)	648(89.9)		
	500 and above	66(7.6)	798(92.4)		
initial ART regimen	Non TDF + 3TC regimen	32(15.1)	180(84.9)	5.26	0.02
	TDF + 3TC regimen	256(10.1)	2285(89.9)		
time on ART	6 months-2 years	163(9.5)	1557(90.5)	8.99	0.03
	3–5 years	63(10.5)	539(89.5)		
	6–8 years	36(14.0)	222(86.0)		
	≥9 years	26(15.0)	147(85.0)		

Further multivariate logistic regression analysis of statistically significant ART outcome variables showed that the risk of VF was 1.96 times higher among men than among women (odds ratio (OR): 1.96, 95% confidence interval (CI): 1.38–2.79); first, CD4^+^ counts were a protective factor for ART, and the higher the CD4+ counts were, the lower the rate of VF, as shown in Table 4.

**Table 4:** Multivariate logistic regression analysis of virological failure

	Variable	Regression coefficient	Standard error	Wald	*P* value	*OR* value(%95*CI*)
Sex	Female					1
	Male	0.67	0.18	14.13	<0.01	1.96(1.38–2.79)
Mode of transmission	Sexual transmission					1
	Injection drugs	−.05	0.16	0.09	0.77	0.95(0.70–1.31)
the first CD4+counts (counts/μl)				12.63	<0.01	
	Below 200					1
	200–349	−1.2	0.19	0.43	0.51	0.89(0.61–1.28)
	350–499	−0.34	0.20	3.02	0.08	0.71(0.49–1.05)
	500 and above	−0.62	0.20	9.67	<0.01	0.54(0.37–0.80)
initial ART regimen	Non-TDF + 3TC regimen					1
	TDF + 3TC regimen	−0.51	0.33	2.39	0.12	0.60(0.32–1.15)
time on ART				3.41	0.33	
	6 months-2 years					1
	3–5 years	0.09	0.16	0.34	0.56	1.10(0.80–1.50)
	6–8 years	0.34	0.20	2.82	0.09	1.41(0.95–2.09)
	≥9 years	−0.17	0.37	0.22	0.64	0.84(0.41–1.74)

### Drug resistance situation

#### Sociodemographic information of drug-resistant patients

Among 2753 AIDS patients who had received ART for more than 6 months, 288 patients were found to have VF, 245 samples were successfully amplified, and the amplification success rate was 85.1% (245/288), of which 111 samples had different degrees of resistance to a certain antiviral drug, and the overall resistance rate in this area was 4.1% (111/2710). Among the 111 resistant patients, more than half were male, the 18–49 age group was predominant, most patients were illiterate, sexual transmission and injection drug transmission accounted for almost half of transmission, and the highest rate of resistance was found among patients with first CD4^+^ counts under 200 counts/μl, with more than 90% of patients on TDF + 3TC as the initial core drug regimen. A total of 68.5% of patients had a change in ART regimen while on medication. The details are shown in Table 5.

**Table 5:** Sociodemographic information of 111 drug-resistant patients

	Characteristic	Number of frequency (cases)	Composition ratio (%)
Sex	Female	34	30.6
	Male	77	69.4
Age group	18–49 years	103	92.8
	50–64 years	8	7.2
Education level	Illiterate	89	80.2
	Elementary school	13	11.7
	Junior high school	8	7.2
	High school or secondary school	1	0.9
Marital status	Unmarried	23	20.7
	Married	80	72.1
	Divorced or widowed	8	7.2
Mode of transmission	Sexual transmission	53	46.8
	Injection drugs	59	53.2
Family type	Single family	38	34.2
	Extended family	37	33.3
	Other	36	32.4
the first CD4+ counts counts/μl	Below 200	36	32.4
	200–349	28	25.2
	350–499	24	21.6
	500 and above	23	20.7
initial ART regimen	Non-TDF + 3TC regimen	10	9.0
	TDF + 3TC regimen	101	91.0
time on ART (years)	6 months-2 years	60	54.1
	3–5 years	27	24.3
	6–8 years	14	12.6
	≥9 years	10	9.0
change of ART regimen	yes	76	68.5
	no	35	31.5

#### Types of drug resistance

**Fig. 1:**
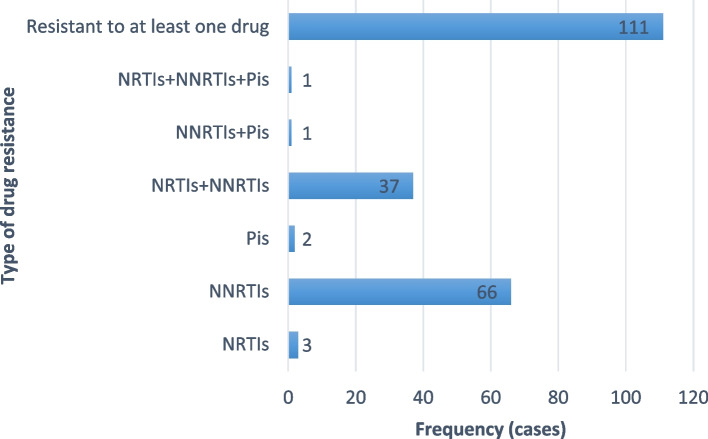
Different types of drug resistance

Among 111 resistant patients, only 3 were found to be resistant to some NRTIs, 66 were found to be resistant to some non-nucleoside reverse transcriptase inhibitors (NNRTIs), and 2 were found to be resistant to some protease inhibitors (PIs); 37 were found to be resistant to both nucleoside reverse transcriptase inhibitors (NRTIs) and NNRTIs, 1 was found to be resistant to both NNRTIs and PIs, and no one was resistant to both NRTIs and PIs; only 1 was found to be resistant to NRTIs, NNRTIs and PIs simultaneously. The information is shown in Fig. 1.

#### Degree of resistance to different drugs

**Fig. 2:**
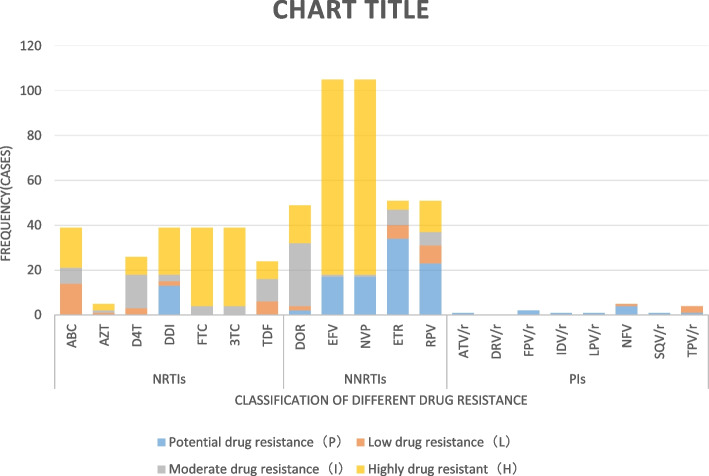
Different drug resistance levels

Among the NRTIs, resistance to 7 different drugs was found, namely, abacavir (ABC), zidovudine (AZT), stavudine (D4T), desoxymesterol (DDI), emtricitabine (FTC), lamivudine (3TC), and tenofovir (TDF), and 39 participants were resistant to both FTC and 3TC, of which 35 were highly resistant and 4 were moderately resistant. Among the NNRTIs, resistance to 5 different drugs was found, namely, doravirine (DOR), efavirenz (EFV), nevirapine (NVP), etravirine (ETR), and rilpivirine (RPV). A total of 105 participants were resistant to both EFV and NVP, of which 87 were highly resistant, 1 was moderately resistant, and 17 were potentially resistant. Among the PIs, resistance to 7 different drugs was found, namely, atazanavir/ritonavir (ATV/r), fosamprenavir/ritonavir (FPV/r), indinavir/ritonavir (IDV/r), lopinavir/ritonavir (LPV/r), nelfinavir (NFV), saquinavir/ritonavir (SQV/r), and tipranavir/ritonavir (TPV/r). Four participants were resistant to both NFV and TPV/r, of which four were potentially resistant to NFV, three had low resistance to TPV/r, and one was potentially resistant to ATV/r. The degree of resistance to different drugs is shown in Fig. 2.

#### Subtype distribution

Among 245 samples, 243 were of the CRF07-BC subtype, accounting for 99.1%, 1 sample was of the CRF01_AE subtype, and 1 sample was of the CRF83-cpx subtype.

#### Drug resistance mutations

**Fig. 3:**
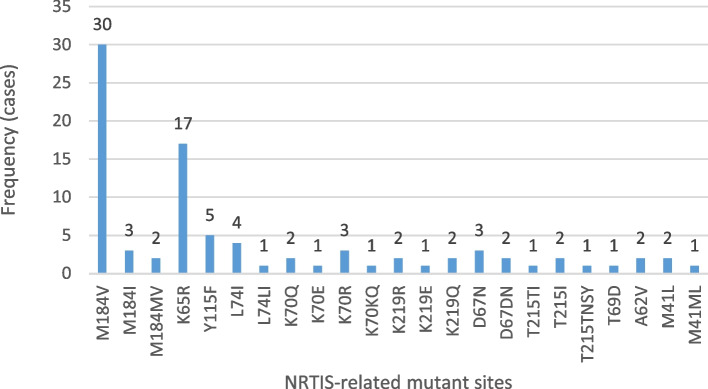
NRTI-related mutation site information

**Fig. 4:**
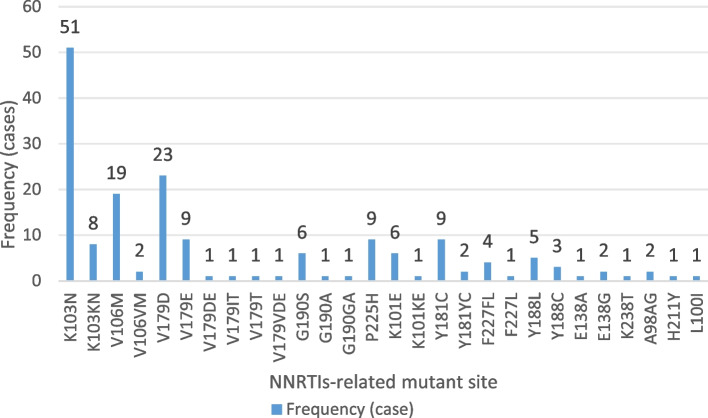
NNRTI-related mutation site information

Among the 245 successfully amplified samples, 23 NRTI-associated drug resistance mutations were identified, of which the most common was the M184V mutation, accounting for 12.2% (30/245), followed by the K65R mutation, accounting for 6.9% (17/245); 28 NNRTI-associated drug resistance mutations were identified, of which the most common was K103N, accounting for 20.8%, followed by the V179D and V106M mutations, accounting for 9.4 and 7.8%, respectively; and 3 PI-associated drug resistance mutation sites were identified, accounting for 9.4 and 7.8%. The most common mutation was K103N, accounting for 20.8%, followed by V179D and V106M, accounting for 9.4 and 7.8%, respectively; three PI-related resistance mutations were identified: one major mutation was M46MIL, and two minor mutations were Q58E and L33LF. The details are shown in Figs. 3 and 4.

### Analysis of the causes of drug resistance

To analyze the possible causes of drug resistance, 245 successfully amplified samples were divided into nonresistant and resistant groups according to whether they were resistant, and analysis by one-way chi-square (χ^2^) test or Fisher’s exact test revealed that patient sex, age group, education level, mode of transmission, family type, initial ART regimen, and time on ART were not related to the occurrence of drug resistance, while patient marital status, first CD4^+^ counts and whether the ART regimen had been changed had a statistically significant effect on drug resistance. The details are shown in Table 6.

**Table 6:** One-way chi-square (χ2) test or Fisher’s exact test for analysis of factors influencing drug resistance

	Variable	Whether drug-resistant (composition ratio)		*χ*^2^	*P value*
		NO	YES		
Sex	Female	30(46.9)	34(53.1)	2.14	0.14
	Male	104(57.5)	77(42.5)		
Age	18–49 years	130(55.8)	103(44.2)	2.32	0.13
	50–64 years	4(33.3)	8(66.7)		
Education level	Illiterate	103(53.6)	89(46.4)	7.48	0.05
	Elementary school	23(63.9)	13(36.1)		
	Junior high school	8(50.0)	8(50.0)		
	High school or secondary school	0	1(100.0)		
Marital status	Unmarried	37(61.7)	23(38.3)	7.87	0.02
	Married	75(48.4)	80(51.6)		
	Divorced or widowed	22(73.3)	8(26.7)		
Mode of transmission	Sexual transmission	51(49.5)	53(50.5)	1.92	0.17
	Injection drugs	83(58.5)	59(53.2)		
Family type	Single family	44(53.7)	38(46.3)	2.32	0.31
	Extended family	35(48.6)	37(51.4)		
	Other	55(60.4)	36(39.6)		
The first CD4+counts (counts/μl)	under 200	24(40.0)	36(60.0)	9.37	0.02
	200–349	30(51.7)	28(48.3)		
	350–499	36(60.0)	24(40.0)		
	500 and above	44(65.7)	23(34.3)		
Initial ART regimen	Non-TDF + 3TC regimen	17(63.0)	10(37.0)	0.84	0.36
	TDF + 3TC regimen	117(53.7)	101(46.3)		
Time on ART	6 months-2 years	81(57.4)	60(42.6)	2.11	0.55
	3–5 years	23(46.0)	27(54.0)		
	6–8 years	16(53.3)	14(46.7)		
	≥9 years	14(58.3)	10(41.7)		
Change of ART regimen	YES	110(59.1)	76(40.9)	6.16	0.01
	NO	24(40.7)	35(59.3)		

Further multifactorial logistic analysis of statistically significant variables for drug resistance revealed that the first CD4^+^ counts were a protective factor against drug resistance, with higher CD4^+^ counts associated with a lower incidence of drug resistance; changing the ART regimen was a risk factor for drug resistance, with the risk of resistance with a change in ART while on medication being 2.10 times higher than without a change (OR: 2.10, 95% CI: 1.12–3.91). The details are shown in Table 7.

**Table 7:** Multifactorial logistic analysis of drug resistance

	Variable	Regression coefficient	Standard error	Wald	*P* value	*OR* value(%95*CI*)
				6.00	0.05	
Marital status	Unmarried					1
	Married	0.43	0.32	1.78	0.18	1.54(0.82–2.89)
	Divorced or widowed	−0.6	0.51	1.42	0.23	0.55(0.20–1.48)
the first CD4+counts (counts/μl)				8.15	0.04	
	Below 200					1
	200–349	−0.43	0.38	1.24	0.27	0.65(0.31–1.38)
	350–499	−0.89	0.39	5.32	0.02	0.41(0.19–0.88)
	500 and above	−0.95	0.38	6.31	0.01	0.39(0.19–0.88)
change of ART regimen	no					1
	yes	0.74	0.32	5.41	0.02	2.10(1.12–3.91)

## Discussion

With the comprehensive development of AIDS prevention and control in remote areas of China and the popularization of HIV ART, HIV drug resistance was bound to emerge, and this will affect the efficacy of HIV treatment; therefore, it is very important to analyze the prevalence of drug resistance to guide the formulation of ART regimens in a timely manner. In this study, the ART efficacy and drug resistance of HIV patients in Meigu County who had been regularly using ART for more than 6 months were analyzed.

In this district, patient sex, mode of transmission, first CD4^+^ counts, initial ART regimen, and time on ART affected the outcome of ART, and further multivariate logistic regression of these factors showed that male sex was a risk factor for VF. The first CD4^+^ counts were protective, and the higher the CD4+ counts were, the lower the VF rate. Possible reasons why men are more likely to fail ART include the following: women have better healthcare behaviors [2]; women have a lower body mass index than men and can maintain higher drug concentration levels in the body and are more likely to achieve virologic suppression [3]; and the presence of adverse behaviors such as smoking in men can affect viral load control [4]. High CD4^+^ counts and high viral load suppression make ART more likely to be successful [5], and the initiation of antiviral therapy in patients with CD4^+^ counts above 500 cells/μl significantly reduces the combined endpoint events of mortality and morbidity [5, 6] and reduces the risk of drug resistance [7], reduced CD4^+^ counts, impaired cellular immune function, and immunity. The more severe the deficiency, the longer the required immune reconstitution takes and the higher the failure rate of ART, based on which it is recommended that ART be initiated rapidly in patients with HIV/AIDS [8, 9].

In this district, 288 cases of ART failure were found, 245 samples were successfully amplified, 111 samples were resistant to a drug, and the viral suppression failure resistance rate was 45.3%, which was higher than the statewide viral suppression failure resistance rates from 2016 to 2019 (25.3, 22.5, 37.7, 35.0%) [10] and lower than the Dazhou 2019 and Guangyuan 2012–2016 virological failure resistance rates [11, 12]. Of the 245 samples successfully amplified in this region, 42.9% of patients were resistant to NNRTIs (105/111), 16.3% were resistant to NRTIs (40/111), and 4.5% were resistant to PIs (5/111). A total of 105 patients showed resistance to both EFV and NVP, of which 87 were highly resistant, and 39 showed resistance to both 3TC and FTC, among whom 35 were highly resistant. In terms of resistance mutations, the most common resistance mutation for NNRTIs was K103N (20.8%, 51/245). In this region, patient marital status, first CD4^+^ counts, and whether the medication was changed during treatment had a statistically significant effect on drug resistance. Further multivariate logistic regression analysis of these factors was conducted, and patients’ first CD4^+^ counts and whether medication was changed during treatment still had an effect on drug resistance. The first CD4^+^ counts was a protective factor against drug resistance; the higher the CD4^+^ counts were, the lower the incidence of drug resistance. Changing the ART regimen was a risk factor for drug resistance. Patients may change regimens due to virological failure, adverse drug reactions, and toxic side effects, and these patients may have risk factors such as poor adherence and missed doses that lead to drug resistance [13].

As of January 1, 2021, the viral suppression rate of adult HIV/AIDS patients in Meigu County who had been on antiviral therapy for more than 6 months was 89.5% (2465/2753), with a low overall resistance rate but a high rate of viral suppression failure [3]. The demographic characteristics of the patients in this study showed that more than half of the patients were male (61.6%, 1697/2753), and the majority of them were aged 18–49 years (94.8%, 2609/2753). It is necessary to focus on young adult male patients in the district because the economic development of the district is poor, and it is common for adult male patients to go out to work, oftentimes in unfixed locations and places, so it is difficult to ensure that these people take oral antiviral drugs on time and in the right amount. This group of patients may have missed doses, poor compliance and other factors that increase the risk of drug resistance. This portion of the population may have a unique sexual situation, and long-term working couples are often separated from each other. There may be high-risk nonmarital sex resulting in the spread of the virus, for which mobile population antiviral management follow-up should be improved. Most of the patients in this district did not attend school, and some studies have shown that those with low literacy and low knowledge of AIDS [14] and poor awareness of prevention will be more likely to be infected with HIV. Thus, it is necessary to strengthen the popularization of AIDS-related knowledge in the population and strengthen compliance among HIV/AIDS patients. Among patients who failed ART and developed drug resistance, EFV, NVP, 3TC, FTC, and DDI resistance were predominant, arising from the fact that most patients’ ART regimen was the nationally recommended first-line ART regimen comprising TDF or AZT + 3TC + EFV/NVP, suggesting that patients who failed ART and developed drug resistance in this district could change to the second-line ART regimen of TDF or AZT + 3TC + LPV/r. There were 4 cases of PI drug resistance in the district and 3 cases of Q58 drug resistance mutation, suggesting that TDR may be in use in the district, and the use of such second-line drugs in the population will increase the difficulty of AIDS prevention and treatment. The district needs to increase drug resistance monitoring and strengthen AIDS prevention and treatment management to prevent the spread of PI drug-resistant strains in the population.

The data on HIV/AIDS patients in this study were obtained from the Chinese Center for Disease Control and Prevention Integrated AIDS Prevention and Control Information System, and no specific demographic survey was conducted, which may have led to information bias affecting the analysis results. In this study, only patients with a viral load ≥1000 copies/ml were tested for drug resistance, but patients with a viral load between 51 and 999 copies/ml may also have drug resistance [15], and more sensitive methods can be explored in future studies to improve the detection rate of drug resistance and achieve the goal of ending the AIDS epidemic by 2030 at an early stage [16].

## Conclusions

The prevention and treatment of AIDS in the district has seen gradual progress, and the overall drug resistance rate is low, but the VF drug resistance rate is higher than the statewide VF drug resistance rate. VF and the occurrence of drug resistance in patients to EFV, NVP, 3TC, FTC, and DDI is dominant because most of the patients have used the first-line antiretroviral regimen TDF or AZT + 3TC + EFV/NVP and 3TC + EFV/NVP, suggesting that patients with VF and drug resistance in this region can instead use second-line antiviral regimens comprising TDF or AZT + 3TC + LPV/r. Integrase strand transfer inhibitors (INSTIs) have become the first choice among antiviral regimens recommended in the international guidelines for the treatment of AIDS [5], and due to the relatively poor economic development of the region, INSTIs have been included in the national health insurance, but few patients and families can afford the expense. If INSTIs are included in the national free antiretroviral program, further progress in AIDS prevention and treatment in this district may be achieved. At the same time, before ART, HIV-1 subtype and drug resistance testing should be carried out to guide the choice of ART to reduce VF and drug resistance, and the effectiveness of ART in the district will be further improved. This study showed that the viral suppression rate in this district is still far from 95%, although the overall viral resistance rate is not high; the viral suppression failure resistance rate is high, mainly due to the drugs in the first-line free program, so increasing the knowledge of AIDS for patients in this district, improving the level of medical care, and strengthening the management of ART and population mobility are still top priorities.

## Data Availability

The datasets used and analyzed during the current study are available from the corresponding author on reasonable request.

## Abbreviations

HIV: Human immunodeficiency virus
AIDS: Acquired immune deficiency syndrome
ART: Antiretroviral therapy
WHO: World health organization
PLWH: People living with HIV
EDTA: Ethylenediaminetetraacetic acid
VL: Viral load
VF: Virological failure
HIVDB: HIV drug resistance database
RT-PCR: Reverse transcription-polymerase chain reaction
CI: Confidence interval
OR: Odds ratios
NNRTI: Nonnucleoside reverse transcriptase inhibitor
NRTI: Nucleoside reverse transcriptase inhibitor
PI: Protease inhibitor
INSTI: Integrase strand transfer inhibitor
TDF: Tenofovir
3TC: Lamivudine
NVP: Nevirapine
DOR: Doravinine
ETR: Etravirine
EFV: Efavirenz
AZT: Zidovudine
LPV/r: Tipranavir/ritonavir
ATV/r: Atazanavir/ritonavir
DRv/r: Darunavir/ritonavir
FPV/r: Tipranavir/ritonavir
IDV/r: Indinavir/Ritonavir
NFV: Nelfinavir
SQV/r: Ritonavir-boosted saquinavir
TPV/r: Tipranavir/ritonavir
ABC: Abacavir
D4T: Stavudine
DDI: Didanosine
FTC: Emtricitabine
ETR: Etravirine
